# Tensile Behaviour of FRCM Composites for Strengthening of Masonry Structures—An Experimental Investigation

**DOI:** 10.3390/ma14133626

**Published:** 2021-06-29

**Authors:** Łukasz Hojdys, Piotr Krajewski

**Affiliations:** Faculty of Civil Engineering, Cracow University of Technology, Warszawska 24, 31-155 Krakow, Poland; piotr.krajewski@pk.edu.pl

**Keywords:** fabric reinforced cementitious matrix, FRCM, textile reinforced mortar, TRM, strengthening system, masonry, tensile test, non-metallic fibres

## Abstract

This paper presents the results of direct tensile tests performed on six different FRCM (fabric reinforced cementitious matrix) strengthening systems used for masonry structures. The emphasis was placed on the determination of the mechanical parameters of each tested system and a comparison of their tensile behaviour in terms of first crack stress, ultimate stress, ultimate strain, cracking pattern, failure mode and idealised tensile stress-strain curve. In addition to the basic mechanical tensile parameters, accidental load eccentricities, matrix tensile strengths, and matrix modules of elasticity were estimated. The results of the tests showed that the tensile behaviour of FRCM composites strongly depends on the parameters of the constituent materials (matrix and fabric). In the tests, tensile failure of reinforcement and fibre slippage within the matrix were observed. The presented research showed that the accidental eccentricities did not substantially affect the obtained results and that the more slender the specimen used, the more consistent the obtained results. The analysis based on a rule of mixtures showed that the direct tensile to flexural tensile strength ratio of the matrixes used in the test was 0.2 to 0.4. Finally, the tensile stress–strain relationship for the tested FRCMs was idealised by a bi- or tri-linear curve.

## 1. Introduction

FRCM (fabric reinforced cementitious matrix) composites are materials composed of structural reinforcing fibre mesh (fabric) embedded in an inorganic matrix. The matrixes for typical FRCM systems for repair and strengthening masonry structures are based on Portland cement, natural hydraulic lime, or geopolymer. Structural reinforcing meshes/grids/fabrics of the composites are made of continuous carbon, basalt, alkali-resistant (AR) glass, PBO (poly-para-phenylene benzobis oxazole) or aramid fibres. There are also some FRCM composites reinforced with steel cords–SRG (steel reinforced grount) systems available on the market. FRCM systems have been widely used for strengthening masonry structures because of their thickness, tensile strength, weight, compatibility with the masonry substrate, vapor permeability, removability, resistance at high temperature, and the ease of their application on wet surfaces [1,2]. Externally bonded FRCM systems have proved to be an effective solution in the strengthening of masonry: columns subjected to vertical loading [3,4,5,6], walls under in-plane loading [7,8,9,10,11], walls under out-of-plane loading [12,13,14,15], vaults and arches [16,17,18,19,20,21,22].

FRCMs have been used mainly to strengthen the tensile zones of masonry structural elements, so the tensile behaviour of FRCM systems has been studied by a number of authors. In [2], FRCMs reinforced with basalt, carbon, and steel textiles were considered. The stress–strain curves observed in the tests were characterized by three stages: uncracked, crack development, and cracked. The authors concluded that the number of textile layers did not affect the ultimate tensile stress and the stiffness of FRCMs reinforced with basalt textile. In [23], De Santis and de Felice tested four ultra-high tensile strength steel reinforced FRCMs combining two textiles and two lime mortars. They noticed that the tensile behaviour of FRCMs was mainly governed by the properties of the textile. Smaller and narrower cracks were observed for more deformable mortar and less dense textile.

The same authors in [24] presented an experimental study of tensile behaviour of FRCMs made with five mortar matrixes and two different textiles (glass-aramid, ultra-high tensile strength steel). In the tests, few clamping methods and testing setups were considered. It was observed that the ultimate strength and the failure mode were mainly governed by the properties of the textile. The contribution of the mortar was nearly negligible for FRCMs reinforced with steel textile and lime mortar. The clamping method influenced the results obtained in the tests. Satisfactory crack patterns and failure modes were observed when additionally reinforced ends of the specimens were clamped directly in the testing machine grips.

Carozzi and Poggi [25] analysed PBO, carbon and glass fibre meshes and three types of cementitious matrixes. The specimens made with PBO fibres were characterized by the highest ultimate strength. It was concluded that clamping the strengthened ends of specimens in testing machine grips was preferable when compared to the clamping method given in the recommendation [26].

Paper [27] presents the results of tensile tests performed on five different FRCM strengthening systems. In this research, PBO, carbon, and glass textiles were used. It was observed that clamping in testing machine grips is the preferred clamping method to obtain a complete characterisation of the composite. Adopting this type of clamping, the research confirmed that FRCM in tension is characterised by a trilinear curve.

Ghiassi et al. [28] tested three different FRCMs reinforced with steel textiles and lime-based and geo-polymeric-based matrixes. Tensile tests showed that tension stiffening behaviour was observed, which was dependent on the mortar mechanical properties.

In [29], the results of tensile tests performed on six different carbon-FRCM strengthening systems are presented. Due to different clamping methods and different properties of the constituents of the systems, not all specimens showed tri-linear behaviour (stress–strain curve). The most common failure mode was cracking of the mortar with rupture of the textile. It was observed that particular attention should be paid in the preparation and curing of the specimens in terms of proper location of the textile, geometry of the sample, and preventing development of microcracks.

Lignola et al. [30] performed tensile tests on four different basalt FRCM systems. The presented results of round robin tensile tests were repeatable in terms of peak stress. Initial elastic behaviour, cracking stress, and stiffness of the specimens were sensitive to the clamping method and pre-cracking at specimen preparation stage.

Paper [31] presents the results of tensile tests performed on specimens of eight different glass FRCM strengthening systems. The authors concluded that not all systems were able to exploit the same tension stiffening effect. During the tests, a large scatter of axial strain was observed, and that the longer gauge length should be used to obtain a better accuracy. Mortar cracking and tensile failure of glass fibre were the most common failure modes. In a few cases, the slippage of the textile was observed. The type of gripping system influenced the failure modes.

De Santis et al. [32] tested four FRCM systems reinforced with unidirectional textiles made with steel cords/ropes. The contribution to the strength and stiffness in the uncracked stage depended on the cord-to-mortar interlocking and the tensile strength of the matrix. The clamping method and proper preparation of the ends of the specimen were crucial to achieve a full stress–strain curve. The ends of the specimen clamped in the testing machine grips should be reinforced to avoid mortar crushing in the grips.

The results of the direct tensile tests performed on one PBO FRCM system and two aramid FRCM systems were discussed in [33]. In the case of the systems reinforced with aramid fabrics bi- or trilinear tensile behaviour was observed. Due to the high density of the aramid textile in one of the tested systems, detachment of two matrix layers from the textile was observed.

In [34], FRCMs consisted of five different types of fabric and three types of mortar were considered. To improve fabric–mortar bond behaviour, polymer coating of fibres was used. The tensile test results confirmed the effectiveness of polymer coating in improving the mechanical properties of FRCMs (ultimate tensile stress and stiffness in the cracked phase). It was found that the contact length of gripping metal tabs of 15 cm was the most suitable for the FRCMs tested in direct tensile tests. Using two or three layers of textile instead of one caused the change in the observed failure mode–the specimens failed due to delamination.

D’Antino and Papanicolaou [35] tested six different composite materials comprising carbon, glass, basalt, or steel textiles which were embedded in lime- or cement-based mortars. The use of fully impregnated textiles resulted in improvement of the mechanical characteristics of FRCMs reinforced with carbon fibres. Misalignment of textile in FRCM specimens led to matrix spalling.

Apart from experimental studies concerning the tensile behaviour of FRCMs, there have been some investigations on analytical or numerical models for FRCMs in tension. In the literature, two approaches can be found: a simplified analytical approach [36,37,38,39] or micro-modelling numerical methods [40,41,42,43].

The above mentioned studies on the tensile behaviour of FRCM materials have shown that the mechanical properties of matrix and fabric, fibre type, fabric architecture, set-up configuration (type of clamping, specimen geometry), as well as matrix–fabric bond properties affect the stress–strain relationship (stiffness, ultimate stress/strain), crack pattern and failure mode of the tested specimens.

To enable the engineering community to use FRCM materials in practice for strengthening masonry structures, American Concrete Institute guides have been developed. They provide recommendations for the design and structural evaluation of externally bonded FRCM systems for repair and strengthening masonry and concrete structures [44,45]. The main mechanical parameters of FRCMs can be determined in direct tensile tests on prismatic specimens according to [26,46]. The bond behaviour can be investigated in shear bond tests according to [47] where a FRCM system is applied on one side of masonry substrate–single-lap shear tests.

The previous studies have shown that, due to the large variability of constituent materials of FRCMs (textile and matrix), each FRCM system for strengthening needs to be tested to determine its unique tensile properties. This paper presents the results of direct tensile tests performed on six different FRCM strengthening systems used for masonry structures. The emphasis was placed on the determination of the mechanical parameters of each tested system and a comparison of their tensile behaviour in terms of first crack stress, ultimate stress, ultimate strain, cracking pattern, failure mode, and idealised tensile stress–strain curve. In addition to the basic mechanical tensile parameters, matrix tensile strengths, and matrix modules of elasticity were estimated based on the results of direct tensile tests of FRCMs. As the results of direct tests are sensitive to load eccentricities, the influence of accidental load eccentricity on the tensile behaviour of FRCM specimens before first crack formation is studied. In the discussion, it is stressed that, for some FRCM materials, the recommendations concerning the construction of an idealised tensile stress–strain curve given in references [26,44,45,48] are not applicable.

## 2. Materials and Methods

### 2.1. Materials

In this research, six commercially available composite external strengthening systems were considered. These systems consist of cement-based or NHL-based matrix and glass, basalt, carbon, or PBO fabric (Table 1).

**Figure 1:**
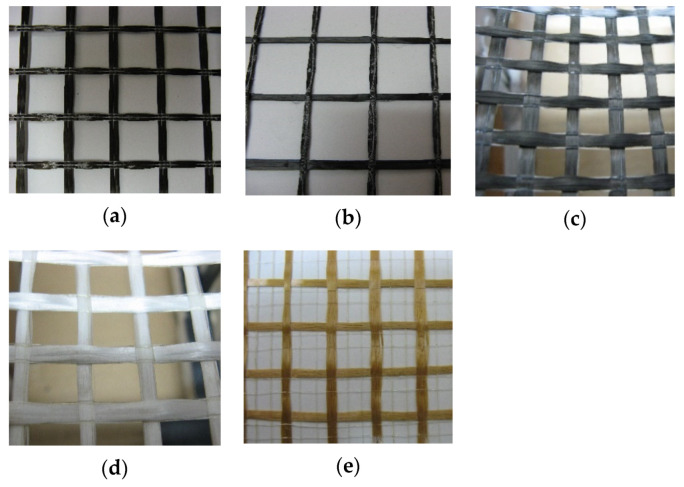
Fabrics used in the test: (**a**) basalt, (**b**) carbon-1; (**c**) carbon-2; (**d**) AR glass; (**e**) PBO.

The fabrics used in the tests are bidirectional grids with the following grid sizes: 25.4 mm—basalt fabric, 30 mm—carbon-1 fabric, 10 mm—carbon-2 fabric, 12 mm—AR glass fabric and 15 mm—PBO fabric (Figure 1). The equivalent thickness of the fabric in one direction is 0.033 mm, 0.060 mm, 0.052 mm and 0.014 mm for basalt, carbon-1, carbon-2, AR glass and PBO fabric, respectively. The basalt fabric and carbon-1 fabric are made with coated yarns whereas the other fabrics are made with uncoated yarns. The mechanical properties of fabrics and matrixes are given in Table 2 and Table 3, respectively. Compressive and flexural strengths of the matrixes given in Table 3 were determined according to EN-1015 11.

### 2.2. Specimen Preparation

**Figure 2:**
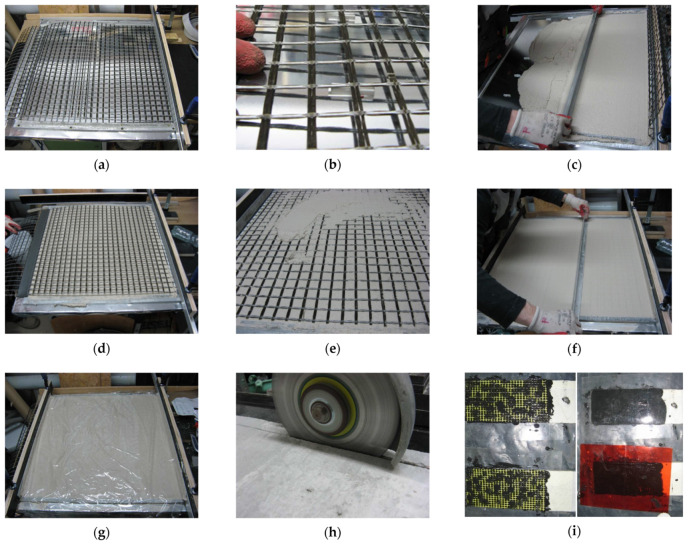
Preparation of the specimens: (**a**) rectangular mould; (**b**) PVC spacer; (**c**) application of the first layer of mortar; (**d**) fabric application; (**e**,**f**) application of the second layer of mortar; (**g**) fresh mortar covered with PVC film; (**h**) cutting out specimens; (**i**) application of the glass grid reinforced polyurethane PS layer on the ends of the specimens.

Prismatic specimens with rectangular cross-sections were prepared (Table 4). The width of the specimens resulted from the grid size of the fabric. It was assumed that the specimen should include at least three yarns and length to width ratio should be at least 5.0 [46]. The fabric was arranged symmetrically with respect to the thickness of the specimen. All specimens, apart from specimens A-C and A-G, were cut out from precast composite plates. In the first stage, for each composite system, a single rectangular mould (approx. 65 cm by 65 cm) was prepared (Figure 2a) with PVC spacers used in order to ensure the correct location of the fabric (Figure 2b). The first layer of mortar was then laid and levelled (Figure 2c). In the next step, the fabric was positioned and slightly prestressed (~3 N per yarn) to straighten the grid and avoid any additional transverse stresses caused by the fabric curvature during the tensile test (Figure 2d). Right after this stage, the second layer of mortar was laid and levelled (Figure 2e,f). The composite plate was then covered with PVC film to minimise early shrinkage of the matrix (Figure 2g). The plate was cured for seven days at 21 ± 2 °C and 95 ± 5% R.H. and then for 21 days at 21 ± 2 °C and 60 ± 10% R.H. After the curing period, the prismatic specimens of the final geometry were cut out from the precast composite plate (Figure 2h). At the final stage of preparation of the specimens, the ends of the specimens were embedded in a polymer (PS) layer reinforced with a glass fibre grid (Figure 2i).

Specimens A-C and A-G were cast by the provider of these strengthening systems and then sent to the laboratory for tests. In this case, each specimen was cast in a single mould. During the preparation of the specimens A-C and A-G, additional layers of primer were applied before and after laying the grid.

### 2.3. Test Set-Up and Testing Procedure

**Figure 3:**
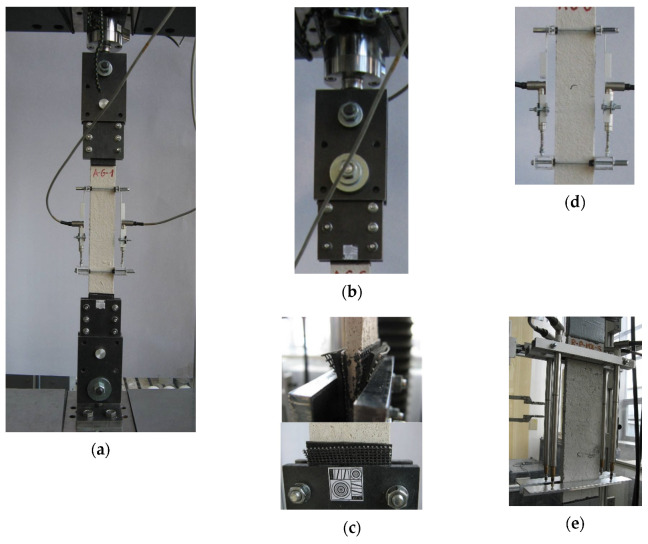
Test set-up: (**a**) general view; (**b**) upper clamp with clevis and force transducer; (**c**) additional layers between the steel plates of the clamping system—2× abrasive mesh, rubber sheet; (**d**) elongation measurement with 2 LVDTs, (**e**) elongation measurement with 4 LVDTs.

Direct tensile tests were performed until failure of the specimens using a universal testing machine (Zwick Z1600, Zwick Roell, Ulm, Germany). Each end of the specimen was clamped with two bolted steel plates (Figure 3a). To avoid slippage at the clamps and to guarantee a homogeneous stress distribution between the steel plate and the specimen, additional layers were used: an abrasive mesh and a rubber sheet (Figure 3c). The steel plates were fixed to the testing machine using a clevis joint with ball hinges (Figure 3b).

The elongation of the central part of the specimens and the applied load were measured during the tests. The base lengths for elongation measurements were: 200 mm for A-C and A-G, 210 mm for B-CTF and B-NHL, 220 mm for C-CTF, and 225 mm for P-MX. Two or four LVDTs (linear variable displacement transducer) attached to the specimens were used for elongation measurement (Figure 3d,e). The applied load was registered with a force transducer HBM U2B 20 kN with a resolution of 0.0012 kN. The load was applied in the displacement control mode with a displacement rate of 0.3 mm/min. in the uncracked stage and 0.5 mm/min. after cracking. All data were acquired with an acquisition frequency of 5 Hz.

## 3. Results

**Figure 4:**
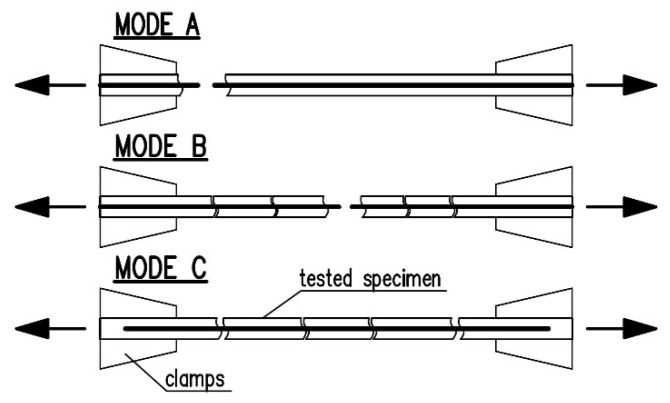
Failure modes of FRCM materials under direct tension.

In this research, 35 direct tensile tests of specimens of six composite strengthening systems were performed. Detailed results are presented in Table 5, Table 6, Table 7, Table 8, Table 9 and Table 10 for first crack stress (σ_t1_), first crack strain (ε_t1_), ultimate stress (σ_u_), strain at failure (ε_u_) and coefficient of variation (CV). The tables also give the exploitation ratio η_Rf_ = σ_u_/f_t_ (where f_t_ is the fabric tensile strength according to Table 2) and the failure modes observed in the tests. The possible failure modes of FRCM materials under direct tension were classified based on specifications in the literature [29,30,31,33] as follows (Figure 4): Mode A—failure at the clamps, Mode B—cracking of the matrix of the specimen with tensile rupture of reinforcement, Mode C—fibre slippage within the matrix.

In this section, the stresses are referred to the cross-sectional area of the reinforcement of the specimen.

### 3.1. B-CTF

**Figure 5:**
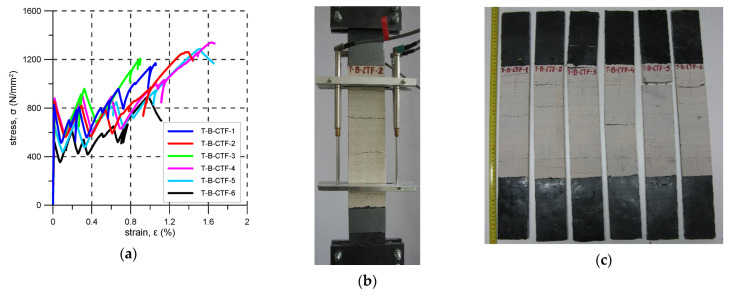
B-CTF test results: (**a**) stress-strain curves; (**b**) crack pattern; (**c**) specimens after the test.

In the case of the B-CTF system, six specimens were tested. As the load was increased, cracks appeared in the mineral matrix. After the first crack formation, a reduction of stiffness was observed. The first crack stress varied from 527 to 880 N/mm^2^ (average 761 N/mm^2^) (Figure 5a, Table 5). Before failure, four to six cracks appeared but only two to four formed inside the elongation base (Figure 5b). Crack spacing varied from 50 mm to 150 mm (mean spacing 79 mm). All specimens failed due to tensile failure of the basalt reinforcement—mode B. The ultimate stresses varied from 899 N/mm^2^ to 1340 N/mm^2^. The average reinforcement exploitation ratio was 0.63. The tensile strength of the fabric given in Table 2 was not reached in any test. For specimens T-B-CTF-1 and T-B-CTF-3, the failure occurred outside of the measurement base of the LVDTs.

### 3.2. B-NHL

**Figure 6:**
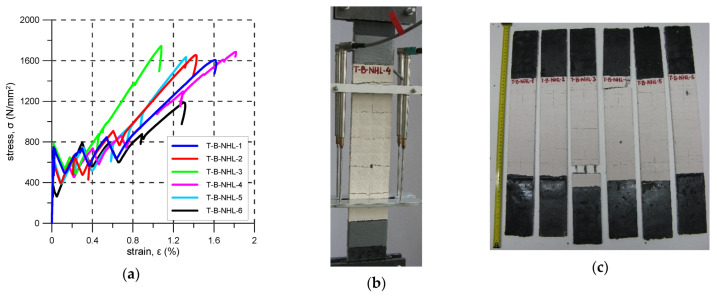
B-NHL test results: (**a**) stress-strain curves; (**b**) crack pattern; (**c**) specimens after the test.

The B-NHL strengthening system consists of a basalt grid embedded in a lime-based matrix. For this system, six specimens were prepared and tested. The first crack formation caused the reduction of stiffness. The first crack stress varied from 370 to 786 N/mm^2^ (average 653 N/mm^2^) (Figure 6a, Table 6). Before failure, four to six cracks appeared but only two to four formed inside the elongation base (Figure 6b). Crack spacing varied from 50 mm to 135 mm (mean spacing 80 mm). Most specimens failed due to fibre slippage–mode C, whereas T-B-NHL-4 failed in a mixed manner (two yarns ruptured and one yarn slipped in the matrix)—mode C/B and in the case of specimen, T-B-NHL-3 rupture of the reinforcement occurred—mode B. The ultimate stress levels in the reinforcement were between 1189 N/mm^2^ and 1744 N/mm^2^. The average reinforcement exploitation ratio was 0.84. Failure of all the specimens occurred outside of the elongation base.

### 3.3. C-CTF

**Figure 7:**
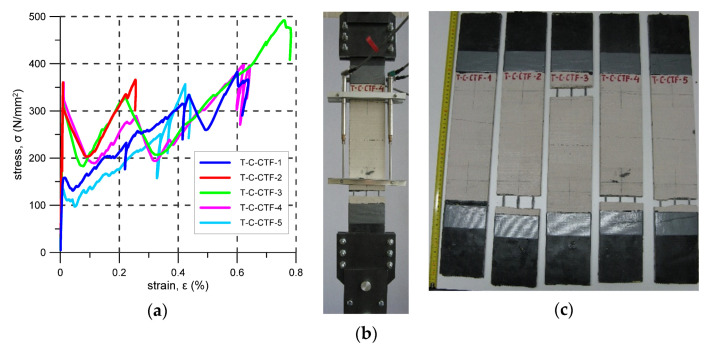
C-CTF test results: (**a**) stress–strain curves; (**b**) crack pattern; (**c**) specimens after the test.

The C-CTF strengthening system consists of cement-based mortar reinforced with a carbon-fibre grid. In this case, five specimens were tested. The first cracks occurred at stress levels from 137 to 360 N/mm^2^ (average 266 N/mm^2^) (Figure 7a, Table 7). Before failure, two to four cracks appeared but only one to two formed inside the elongation base (Figure 7b). Crack spacing varied from 75 mm to 220 mm (mean spacing 124 mm). All specimens failed due to fibre slippage—mode C. The ultimate stress was between 357 N/mm^2^ and 492 N/mm^2^ and the average reinforcement exploitation ratio was 0.22. Failure of all the specimens occurred out of the elongation base, near the steel clamps.

### 3.4. A-C

**Figure 8:**
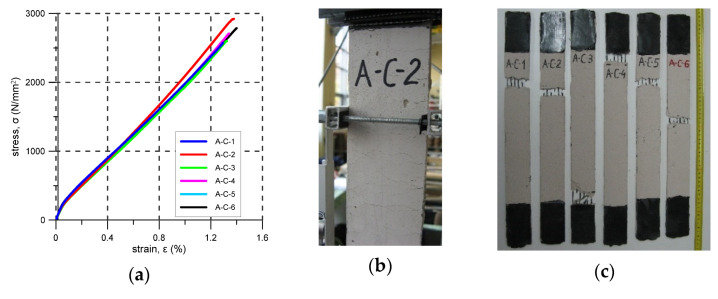
A-C test results: (**a**) stress–strain curves; (**b**) typical crack pattern; (**c**) specimens after the test.

Six prismatic specimens of A-C composite were tested. The A-C system consists of lime-based mortar and carbon grid reinforcement covered with primer applied during the preparation of the specimens. The first cracks were noticed (were visible) at stresses between 390 N/mm^2^ and 600 N/mm^2^, but based on the σ-ε plots presented in Figure 8a, the loss of linearity occurred for a stress of about 150 N/mm^2^. The whole specimens were uniformly cracked over their length. Crack spacing was about 5–20 mm–map/pattern cracks were observed, see Figure 8b. All specimens failed due to tensile failure of the carbon reinforcement—mode B. The ultimate stress varied from 2225 N/mm^2^ to 2922 N/mm^2^ (Table 8). The average reinforcement exploitation ratio was 0.78. For the specimens A-C-3 and A-C-4, fibre tensile failure occurred near the steel clamps outside of the elongation base.

### 3.5. A-G

**Figure 9:**
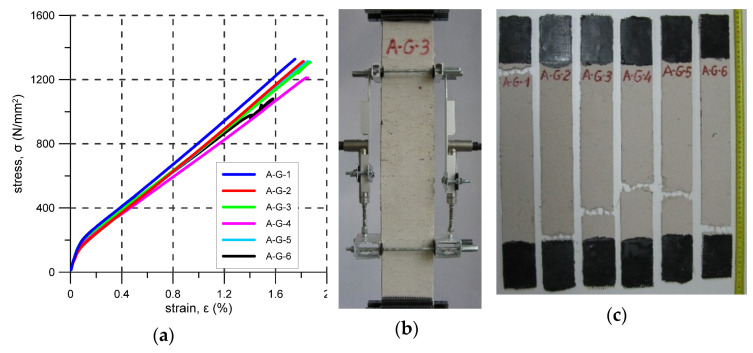
A-G test results: (**a**) stress–strain curves; (**b**) typical crack pattern; (**c**) specimens after the test.

The A-G system is similar to the A-C system but instead of a carbon fibre grid, an AG glass fibre grid is used as reinforcement. For this system, six specimens were prepared and tested. The first cracks were noticed (were visible) at stress levels not less than 280 N/mm^2^, but based on the σ-ε plots presented in Figure 9a, a loss of linearity of around 110 N/mm^2^ occurred for lower stresses. During the test, map/pattern cracks were observed (Figure 9b). Crack spacing was about 5–20 mm. All specimens failed due to the tensile failure of glass reinforcement–mode B. The ultimate stress varied from 1081 N/mm^2^ to 1329 N/mm^2^ (Table 9). The average reinforcement exploitation ratio equals 0.90. For the specimen fibres A-G-1, A-G-2, A-G-3 and A-G-6, tensile failure occurred near the steel clamps outside the elongation base (Figure 9c). During the test of specimens A-G-6, slippage of the specimen in the lower clamp occurred. The test was stopped and, after the bolts were refastened, the test was continued.

### 3.6. P-MX

**Figure 10:**
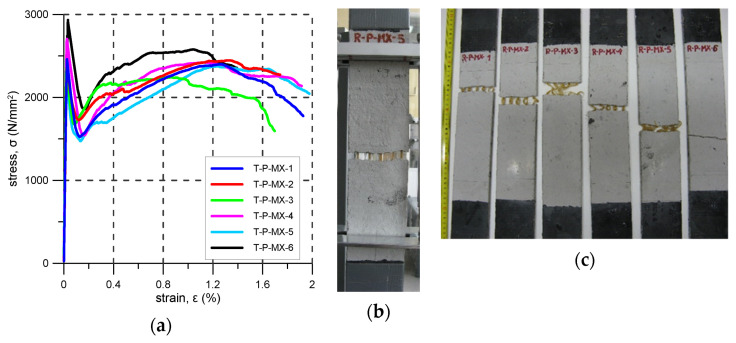
P-MX test results: (**a**) stress–strain curves; (**b**) crack pattern; (**c**) specimens after the test.

The P-MX strengthening system consists of a PBO grid embedded in a cement-based matrix. For this system, six specimens were tested. The first and only crack formed at stress between 2235 and 2932 N/mm^2^ (average 2516 N/mm^2^) (Figure 10a,b, Table 10). All specimens failed due to fibre rupture—mode B. The maximum residual stress (σ_R_) in the reinforcement was between 2244 N/mm^2^ and 2578 N/mm^2^. The average reinforcement exploitation ratio was 0.76. Failure of all the specimens occurred inside the elongation base.

Due to the first crack development at a stress level of σ_t1_, a rapid drop of tensile stress was observed (ca. 30%). After the first crack formation, strain hardening behaviour was noticed, and the tensile stress increased up to the residual tensile strength (σ_R_) (see Figure 10a and Table 10).

## 4. Discussion

In this research, six FRCM strengthening systems for masonry structures were considered. Adopted fabrics varied in terms of the type of fibre, architecture and tensile strength, and they were embedded in one of four different matrixes. The differences between the properties of the materials used in the experiments strongly influenced the tensile behaviour of the tested specimens. The main results of tensile tests are presented in Table 5, Table 6, Table 7, Table 8, Table 9 and Table 10. It could be noted that CVs (coefficients of variation) for most results were not greater than 20%, which indicates that the results are consistent. Only for the C-CTF specimens was a higher dispersion of results observed—CV up to 40%, which could be explained by the geometry of the specimen—for the lowest value of length/width ratio, see Table 4. It can be concluded that the more slender the specimen, the more consistent the obtained results (for the most slender specimens A-G, A-C, CV were less than 10%).

In Table 11, a comparison between the tensile strength of composite (f_uc_) and fabric (f_uf_) per unit of width is presented. The tensile strength of FRCM varied between 23 kN/m and 137 kN/m for C-CTF and A-C, respectively. As the fabric tensile strength is the main factor affecting composite tensile strength, it was expected that systems with reinforcement of the highest fabric strength (f_uf_) (carbon fibre fabrics) would be the most efficient option in terms of composite tensile strength (f_uc_). However, it turned out that the C-CTF composite (system with fabric with the second tensile strength) was characterised by the lowest value of both composite tensile strength and exploitation ratio. Typical values of exploitation ratio in the tests varied from 0.6 to 0.9. In the case of C-CTF, this was 0.22. Such a low value was probably the effect of fabric architecture. This fabric was characterised by low dispersion of carbon reinforcement (large spacing between the bundles, high concentration of stiff fibres in a single bundle), which affected the fabric–matrix bond behaviour and resulted in bundle slippage within the matrix (failure mode C).

**Figure 11:**
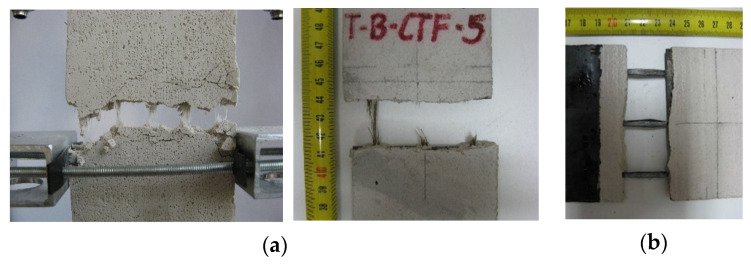
Failure modes observed in the tests: (**a**) fabric rupture—Mode B (specimens A-G-6 and B-CTF-5); (**b**) fabric slippage—Mode C (specimen C-CTF-2).

In the tests, two types of failure mode were observed: mode B-tensile reinforcement rupture; mode C-fibre slippage inside the matrix (Figure 11) (failure mode classification was adopted based on specifications reported in the literature [29,30,31,33]). Most of the specimens failed due to fabric rupture (mode B). In the case of composite C-CTF and two specimens of B-NHL, slippage of bundles within the matrix was observed (mode C). This type of failure was also observed by other authors, especially when a clamping system with bolted steel plates was used [24,29,30,31,32,33]. This failure mode was caused by the low bond of bundles to the matrix. To provide adequate anchorage length for the fabric, one of the following modifications of the testing set-up could be made: higher pressure in the clamps obtained through the use of pneumatic or hydraulic gripping; use of a longer specimen together with longer bolted steel plates of the clamping system; lengthening the specimen outside the bolted steel plates of the clamping system to provide adequate reinforcement anchorage length.

In Figure 5b, Figure 6b, Figure 7b, Figure 8b, Figure 9b and Figure 10b typical crack patterns observed in the test are presented. The cracks developed perpendicularly to the longitudinal axis of the specimens apart from specimens A-C and A-G where map/pattern cracking was observed. The final (just before the failure) number of cracks and their spacing varied. In the case of composite P-MX, only one crack was observed—this is typical for composites with a reinforcement ratio that is too low. Before the tests, it was estimated that the cross-sectional area of the PBO reinforcement fulfilled the minimum reinforcement requirement. The observed behaviour of the P-MX specimens and the results given in Table 10 showed that assuming η_Rf_ = 0.76, the minimum cross-sectional area of PBO fabric should be around 1.13 mm^2^ in each specimen (compare with the data in Table 4).

For B-CTF, B-NHL, and C-CTF, cracks developed at the location of the bundles in the weft direction, so the distance between them was close to two, three, or four times the distance between bundles. As the final distance between cracks is related to the bond strength of the bundles, the greatest distance between cracks observed for C-CTF indicates the poorest bond between carbon fibre bundles and the CTF matrix when compared to the other systems. On the other hand, the crack pattern (map cracking) observed for A-C and A-G composites indicates a good bond between the fabric and matrix. This could result from the application of two extra layers of primer to the fabric when these specimens were cast.

The tensile behaviour of the composites in the first stage (before cracking) is governed by the tensile parameters of the matrix. Based on the results of tensile tests of composites, the direct tensile strength and tensile modulus of elasticity of the matrixes were computed. In the calculation, the following assumptions were made: the plane sections remain plane; the linear stress–strain relation for matrix and fabric; the strain in bonded reinforcement is the same as that in the surrounding matrix; uni-directional reinforcement; the rule of mixtures for stiffness is valid—see Equation (1), where E_c_, E_m_, E_f_—composite, matrix and fabric elastic modulus, respectively; V_m_, V_f_—volume fraction of matrix and fabric.

E_c_ = E_m_V_m_ + E_f_V_f_, (1)

Mechanical parameters and geometry assumed in the analysis were taken from Table 2 and Table 4. In this analysis, the following parameters of the matrix were estimated: elastic modulus (E_m_), tensile strength taking into account accidental load eccentricity (f_t_), tensile strength assuming axial loading (f′_t_)—Table 12. As tensile strains were measured using two to four LVDTs, it was possible to estimate the eccentricity of the load applied to the specimen. The value of eccentricity (e_m_) in relation to the width of the specimen (b) was from 0.9% to 5.1%, so this indicates that the specimens were almost axially loaded. The accidental eccentricities did not substantially affect the computed values of the tensile strength of the matrix. The maximum difference between the tensile strength of the matrix was computed assuming that the axial and eccentrical load is up to 15% for C-CTF. The tensile strengths of a matrix (f_t_, f′_t_), computed as assumed above, were compared with the tensile strength of the matrix in bending (f_fl_). The flexural tensile strength was determined in tests on 40 × 40 × 160 mm^3^ mortar specimens. The comparison was made for specimens of the same age—42 days. The f_t_/f_fl_ and f′_t_/f_fl_ ratios are presented in Table 12. It can be observed that the computed tensile strengths (under quasi-axial load) are ca. 20% to 40% of the flexural strength of matrix—this is a typical relationship between the direct and flexural strengths of brittle cementitious materials [49]. Tensile strength of the matrix of composites A-C and A-G was not computed because the moment of the first crack formation was not noticeable.

The stiffnesses of the matrixes used in the research were similar (elastic modulus from 13.8 kN/mm^2^ to 16.6 kN/mm^2^) apart from for matrix type A which was used in composites A-C and A-G. In this case, the modulus of elasticity was ca. 2 kN/mm^2^. The modules of elasticity, computed on the basis of the data from laboratory direct tensile tests of composites (Table 12), are greater than the elastic modules declared by the manufacturers (Table 3). In the case of P-MX composite, the computed elastic modulus was two times greater than specified by the manufacturer in the datasheet and this corresponds to the declared vs. tested compressive strength ratio (around 2.0). For composite B-NHL, the computed elastic modulus is close to the value declared by the manufacturer.

**Figure 12:**
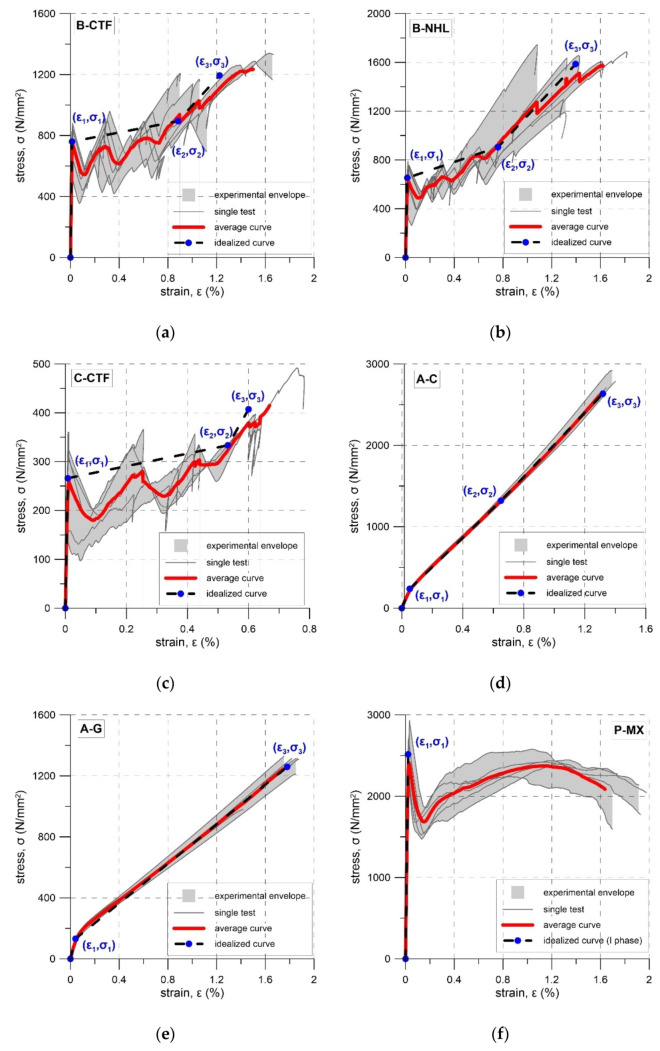
Idealised stress-strain curves: (**a**) B-CTF; (**b**) B-NHL; (**c**) C-CTF; (**d**) A-C; (**e**) A-G; (**f**) P-MX.

During tensile tests of an FRCM, three stages can be distinguished: I stage–uncracked, II stage—crack development, III cracked stage [29,30,31,32,33]. Stress–strain curve responses recorded in the tests are presented in Figure 5, Figure 6, Figure 7, Figure 8, Figure 9, Figure 10, Figure 11 and Figure 12. In the case of B-CTF, B-NHL, and C-CTF at the moment of each crack formation, a rapid drop in stress was recorded, so the beginning of the second stage was easy to determine. Stress–strain curves for composites A-C and A-G are smooth and it is difficult to precisely indicate the transition points between adjacent stages for these materials. As the minimum reinforcement ratio for P-MX composite was not provided, only one crack developed in the tests. In this case, two stages were observed: an uncracked stage and a cracked stage.

In the design of FRCM strengthening for masonry structures, the experimental stress–strain curves can be idealised in accordance with specifications in the literature [26,44,45,48]. The idealised curves for the tested specimens are presented in Figure 12 and the parameters of the idealised curves are given in Table 13. The tensile behaviour of B-CTF, B-NHL and C-CTF can be idealised by the trilinear curve [48] (Figure 12a–c). As the moments of both the first crack appearance and crack development were not clear, the recommendations given in the literature [26,45] were used to define the idealised bilinear curve for composite A-G (Figure 12e). In the case of A-C composite, neither of the aforementioned recommendations could be used, so the following approach was proposed: the branch in the first stage was to be characterised by the modulus of elasticity E_1_ calculated as the slope of the regression line for the initial, linear part of the experimental stress–strain relation; the modulus E_2_ would then be calculated as the slope of a line that connects two points at a stress level equal to 0.1σ_u_ and 0.5σ_u_; modulus E_3_ was then to be calculated as a slope of a line that connects two points at stress levels of 0.5σ_u_ and 0.9σ_u_. Transition points (σ_1_, ε_1_) and (σ_2_, ε_2_) correspond to the intersection points obtained by continuing the initial and the second branch, and the second and the third branch of the idealised curve, respectively. The coordinates of the last point of the third linear branch (σ_3_, ε_3_) were calculated for the stress level σ_3_ = σ_u_ (Figure 12d). As for the P-MX composite, no strain hardening post-cracking behaviour was observed, and an idealised linear stress-strain curve up to the first crack formation was proposed (Figure 12f).

The B-CTF and B-NHL composites were reinforced with an identical basalt fabric, but they differed in the matrix type. Comparing the behaviour of these systems (Figure 12a,b), the following similarities can be seen: the first crack was observed at a strain of about 0.013%, the cracking development stage was up to a stress level of around 900 N/mm^2^, and the stiffness in stage III was close to the stiffness of the fabric. The main differences in the behaviour of these composites were in the failure modes, ultimate stresses, and strains. These may result from the difference in bundle-matrix bond and the difference in the size of load eccentricities in the tests (Table 12).

## 5. Conclusions

The results of direct tensile tests performed on FRCM systems for strengthening masonry structures have been presented in this paper. The main conclusion can be summarised as follows:

- The tensile behaviour of FRCM composites strongly depends on the parameters of the constituent materials (matrix and fabric). The tensile properties of each type of FRCM should be determined in a direct tensile test in accordance with available recommendations and guides [26,44,45,46].
- It is suggested that the specimens for the direct tensile testing of FRCMs should have a length to width ratio of not less than 5.0. The presented research shows that the more slender the specimen used, the more consistent the obtained results.
- Typical values of the reinforcement exploitation ratio in the tests varied from 0.6 to 0.9. When a fabric was characterised by a large spacing between the bundles and high concentration of stiff fibres in a single bundle, the exploitation ratio dropped to 0.22 (C-CTF composite).
- In the tests, tensile failure of reinforcement (mode B) and fibre slippage within the matrix (mode C) were observed. If the majority of specimens fail due to fibre slippage, a modification of the testing set-up should be considered: higher pressure in the clamps, longer specimen and longer bolted steel plates of the clamping system or lengthening of the specimen outside the bolted steel plates of the clamping system.
- The amount of reinforcement adopted in the FRCM system should provide strain hardening post-cracking behaviour. This requirement was not fulfilled for the P-MX composite, so in this case the FRCM tensile strength was equal to the first crack stress.
- Tensile strengths and modules of elasticity of the matrixes used in the test were computed using a rule of mixtures equation. It could be observed that the direct tensile to flexural tensile strength ratio was 0.2 to 0.4. The stiffnesses of the matrixes used in the research were from 13.8 kN/mm^2^ to 16.6 kN/mm^2^ for B-NHL, B-CTF, C-CTF, P-MX composites and ca. 2 kN/mm^2^ for A-C and A-G composites.
- It is suggested to use at least two gauges to measure tensile strain. This allows estimation of the eccentricity of the load applied to the specimen. In the presented tests, the accidental eccentricities did not substantially affect the obtained results.
- The tensile stress–strain relationship for FRCMs can be idealised by a bi- or trilinear curve. In this paper, idealised curves for the tested materials were suggested.

## Figures and Tables

**Table 1:** FRCM systems used in experimental tests.

FRCM System	Fabric	Mortar
B-CTF	basalt	CTF–cement-based one-component mortar
B-NHL	basalt	NHL–cement-free NHL-based one-component mortar ^1^
C-CTF	carbon-1	CTF–cement-based one-component mortar
A-C	carbon-2	A–NHL-based mortar ^2^
A-G	AR glass	A–NHL-based mortar ^2^
P-MX	PBO	MX–cement-based mortar

^1^ NHL—cement-free, one-component mortar with high pozzolanic action composed of natural hydraulic lime, graded sand (<3 mm), pozzolanic binders, and synthetic fibers. ^2^ A–Brigliadori fibre-reinforced NHL based mortar plus Primer IPN 01.

**Table 2:** Mechanical properties of fabrics used in the tests.

basalt ^1^	
tensile strength	1900 N/mm^2^
modulus of elasticity	107,000 N/mm^2^
carbon-1 ^2^	
tensile strength	1800 N/mm^2^
modulus of elasticity	227,000 N/mm^2^
carbon-2 ^3^	
tensile strength	3400 N/mm^2^
modulus of elasticity	240,000 N/mm^2^
AR glass ^3^	
tensile strength	>1400 N/mm^2^
modulus of elasticity	74,000 N/mm^2^
PBO ^4^	
tensile strength	3356 N/mm^2^
modulus of elasticity	191,600 N/mm^2^

^1^ single roving test, according to [30,35], ^2^ according to [29], ^3^ specified by manufacturer, ^4^ according to [33].

**Table 3:** Mechanical properties of mortars (matrixes) used in the tests at 28 days.

CTF	
compressive strength	15.4 N/mm^2^
tensile strength (flexural)	5.0 N/mm^2^
modulus of elasticity	N/A
NHL	
compressive strength	14.9 N/mm^2^
tensile strength (flexural)	5.4 N/mm^2^
modulus of elasticity ^1^	10000 N/mm^2^
A ^2^	
compressive strength	9.8 N/mm^2^
tensile strength (flexural)	3.8 N/mm^2^
modulus of elasticity	N/A
MX	
compressive strength	44.3 N/mm^2^
tensile strength (flexural)	9.3 N/mm^2^
modulus of elasticity ^1^	7500 N/mm^2^

^1^ specified by manufacturer, ^2^ according to [29], N/A—not available.

**Table 4:** Geometry of the specimens.

FRCM System	Length (mm)	Thickness (mm) *	Width (mm) *	Length/Width Ratio (–)	Number of Yarns	Fabric Cross Section (mm^2^)
B-CTF	585	10/11.6	75/77	7.7	3	2.49 ^1^
B-NHL	585	10/11.8	75/77	7.7	3	2.49 ^1^
C-CTF	595	10/11.4	94/96	6.2	3	5.67 ^2^
A-C	500	6/6.8	54/55	9.2	6	2.82 ^3^
A-G	500	6/6.5	60/61	8.2	6	3.60 ^3^
P-MX	590	10/11	75/76	7.8	5	1.05 ^4^

* Min/max. recorded deviations (if any), ^1^ dry basalt fibres, according to [30,35], ^2^ according to [29], ^3^ specified by the manufacturer, ^4^ according to [33].

**Table 5:** B-CTF test results.

Test	σ_t1_	ε_t1_	σ_u_	ε_u_	η_Rf_	Failure Mode
	(N/mm^2^)	(%)	(N/mm^2^)	(%)	(–)	
1	825	0.011	1169	1.06	0.62	B
2	853	0.015	1262	1.39	0.66	B
3	818	0.013	1208	0.90	0.64	B
4	880	0.017	1340	1.64	0.71	B
5	660	0.013	1288	1.51	0.68	B
6	9527	0.015	899	0.98	0.47	B
Average	761	0.014	1194	1.25	0.63	
CV (%)	18.1	15.0	13.1	24.6	13.4	

**Table 6:** B-NHL test results.

Test	σ_t1_	ε_t1_	σ_u_	ε_u_	η_Rf_	Failure Mode
	(N/mm^2^)	(%)	(N/mm^2^)	(%)	(–)	
1	749	0.017	1606	1.61	0.85	C
2	609	0.015	1655	1.42	0.87	C
3	786	0.020	1744	1.08	0.92	B
4	759	0.014	1686	1.82	0.89	B/C
5	645	0.017	1635	1.32	0.86	C
6	370	0.013	1189	1.30	0.63	C
Average	653	0.016	1586	1.43	0.84	
CV (%)	23.7	15.8	12.6	18.2	12.5	

**Table 7:** C-CTF test results.

Test	σ_t1_	ε_t1_	σ_u_	ε_u_	η_Rf_	Failure Mode
	(N/mm^2^)	(%)	(N/mm^2^)	(%)	(–)	
1	158	0.013	383	0.60	0.21	C
2	360	0.009	366	0.25	0.2	C
3	315	0.009	492	0.76	0.27	C
4	360	0.011	397	0.62	0.22	C
5	137	0.007	357	0.42	0.2	C
Average	266	0.010	399	0.53	0.22	
CV [%]	41.3	23.3	13.6	37.3	13.3	

**Table 8:** A-C test results.

Test	σ_t1_	ε_t1_	σ_u_	ε_u_	η_Rf_	Failure Mode
	(N/mm^2^)	(%)	(N/mm^2^)	(%)	(–)	
1	-	-	2575	1.29	0.76	B
2	-	-	2922	1.38	0.86	B
3	-	-	2601	1.32	0.76	B
4	-	-	2708	1.34	0.8	B
5	-	-	2225	1.13	0.65	B
6	-	-	2787	1.40	0.82	B
Average	-	-	2636	1.31	0.78	
CV [%]	-	-	9.0	7.4	9.3	

**Table 9:** A-G test results.

Test	σ_t1_	ε_t1_	σ_u_	ε_u_	η_Rf_	Failure Mode
	(N/mm^2^)	(%)	(N/mm^2^)	(%)	(–)	
1	-	-	1329	1.75	0.95	B
2	-	-	1314	1.82	0.94	B
3	-	-	1307	1.87	0.93	B
4	-	-	1212	1.84	0.87	B
5	-	-	1312	1.85	0.94	B
6	-	-	1081	1.58	0.77	B
Average	-	-	1259	1.79	0.90	
CV (%)	-	-	7.7	6.1	7.8	

**Table 10:** P-MX test results.

Test	σ_t1_	ε_t1_	σ_R_	ε_R_	η_Rf_	Failure Mode
	(N/mm^2^)	(%)	(N/mm^2^)	(%)	(–)	
1	2460	0.025	2401	1.27	0.73	B
2	2422	0.025	2466	1.31	0.73	B
3	2342	0.020	2244	0.86	0.70	B
4	2706	0.023	2421	1.13	0.81	B
5	2235	0.025	2377	1.23	0.71	B
6	2932	0.029	2578	1.04	0.87	B
Average	2516	0.025	2415	1.14	0.76	
CV (%)	10.2	12.0	4.5	14.8	8.8	

**Table 11:** Tensile strength of composite (f_uc_) and fabric (f_ut_).

FRCM System	f_uc_	f_uf_	η_Rf_	Failure Mode
	(kN/m)	(kN/m)	(–)	
B-CTF	40	63	0.63	B
B-NHL	53	63	0.84	B/C
C-CTF	23	106	0.22	C
A-C	137	178	0.78	B
A-G	75	84	0.90	B
P-MX	36	47	0.76	B

**Table 12:** Computed matrix parameters.

FRCM System	E_m_	e_m/_b	f_t_	f′_t_	f_fl_	f_t_/f_fl_	f′_t_/f_fl_
	(kN/mm^2^)	(%)	(N/mm^2^)	(N/mm^2^)	(N/mm^2^)	(–)	(–)
B-CTF	16.6	2.4	2.3	2.1	5.8	0.40	0.36
B-NHL	13.8	0.9	1.8	1.7	6.7	0.27	0.25
C-CTF	15.7	5.1	1.5	1.3	6.4	0.23	0.20
A-C	1.8	2.0	-	-	-	-	-
A-G	2.4	1.1	-	-	-	-	-
P-MX	14.8	1.5	3.5	3.2	11.0	0.32	0.29

**Table 13:** ACI and RILEM idealised curves parameters.

FRCM System	E_1_	σ_1_	ε_1_	E_2_	σ_2_	ε_2_	E_3_	σ_3_	ε_3_
	(kN/mm^2^)	(N/mm^2^)	(%)	(kN/mm^2^)	(N/mm^2^)	(%)	(kN/mm^2^)	(N/mm^2^)	(%)
B-CTF	6060	761	0.013	15	895	0.88	91	1194	1.22
B-NHL	4749	653	0.014	33	905	0.76	108	1586	1.40
C-CTF	3190	266	0.008	16	339	0.48	116	399	0.53
A-C	459	237	0.052	182	1318	0.65	197	2636	1.32
A-G	317	132	0.042	-	-	-	65	1259	1.78
P-MX	11722	2516	0.022	-	-	-	-	-	-

## Data Availability

The data presented in this study are available on request from the corresponding author.
